# CVMFusion: ConvNeXtV2 and Visual Mamba Fusion for Remote Sensing Segmentation

**DOI:** 10.3390/s26020640

**Published:** 2026-01-18

**Authors:** Zelin Wang, Li Qin, Cheng Xu, Dexi Liu, Zeyu Guo, Yu Hu, Tianyu Yang

**Affiliations:** 1 National Key Laboratory of Complex System Control and Intelligent Agent Cooperation, Beijing 100074, China; wzl162601@outlook.com (Z.W.); 3190101923@zju.edu.cn (Y.H.); 2 Beijing Research Institute of Telemetry, Beijing 100076, Chinayangty2001@outlook.com (T.Y.)

**Keywords:** Mamba, convolutional neural network, remote sensing, image segmentation, attention mechanisms

## Abstract

**Highlights:**

**What are the main findings?**

- This paper presents CVMFusion, an innovative dual-branch network that cohesively combines ConvNeXtV2 for precise local feature extraction and VMamba for extensive contextual modelling, therefore setting a new benchmark for sea–land segmentation in remote sensing data.
- The proposed Dynamic Multi-scale Attention (DyMSA) and Dynamic Weighted Cross-Attention (DyWCA) modules enable dynamic, adaptive feature fusion, which is empirically shown to enhance the segmentation accuracy of small targets and complex coastline boundaries.

**What are the implications of the main findings?**

- The exceptional performance of CVMFusion on public SAR datasets illustrates the effectiveness of the hybrid CNN-Mamba architecture in addressing the shortcomings of current approaches, especially in managing class imbalance and retaining essential edge information.
- This work provides a robust and accurate tool for coastal zone monitoring, with direct implications for improving applications in marine disaster early warning, navigation safety, and sustainable coastal resource management.

**Abstract:**

In recent years, extracting coastlines from high-resolution remote sensing imagery has proven difficult due to complex details and variable targets. Current methods struggle with the fact that CNNs cannot model long-range dependencies, while Transformers incur high computational costs. To address these issues, we propose CVMFusion: a land–sea segmentation network based on a U-shaped encoder–decoder structure, whereby both the encoder and decoder are hierarchically organized. This architecture integrates the local feature extraction capabilities of CNNs with the global interaction efficiency of Mamba. The encoder uses parallel ConvNeXtV2 and VMamba branches to capture fine-grained details and long-range context, respectively. This network incorporates Dynamic Multi-Scale Attention (DyMSA) and Dynamic Weighted Cross-Attention (DyWCA) modules, which replace the traditional concatenation with an adaptive fusion mechanism to effectively fuse the features from the dual-branch encoder and utilize skip connections to complete the fusion between the encoder and decoder. Experiments on two public datasets demonstrate that CVMFusion attained MIoU accuracies of 98.05% and 96.28%, outperforming existing methods. It performs particularly well in segmenting small objects and intricate boundary regions.

## 1. Introduction

Coastal regions, defined by the continuous interaction between land and sea, experience persistent alterations in their geography. These alterations result from both natural processes and human activity [1]. Precise monitoring of these changing boundaries, delineated by the separation of water and land, is essential for numerous significant applications. These include early warning systems for disasters, navigation safety measures, and the proper management of coastal resources [2,3,4]. Remote sensing is an essential technique for this objective, providing extensive, all-weather, broad-area, and prompt observations, albeit producing a significant volume of data. Conventional techniques for separating land and water mostly rely on thresholding approaches that use spectral data [5,6]. These methods define land areas by exploiting contrasts in brightness and texture between land and water. Yet, they struggle to differentiate between features that appear identical in the spectrum. As a result, the chosen thresholds are vulnerable to image noise and fluctuations in scattering mechanisms [7,8]. As a result, it is replacing conventional methods and has emerged as a significant area of research. Convolutional Neural Networks (CNNs) are widely employed in image segmentation, resulting in numerous architectures such as R-CNN [9] and DenseNet [10]. Nevertheless, CNNs are inherently limited by their small receptive fields, which restricts their ability to capture long-range dependencies and contextual information over extensive regions.

The self-attention mechanism inherent in the Transformer architecture [11] solves this limitation by effectively modeling long-term dependencies and realizing global feature aggregation. Therefore, this innovation has led to the widespread use of Transformers and their variants in image segmentation applications. As an illustration, Ai et al. [12] developed an AIS-assisted Pyramid Vision Transformer (AIS-PVT) for sea–land segmentation, leveraging multi-scale global information to enhance data fusion capabilities. Tong et al. [13] proposed the STIRUnet model, which combines the global modeling capability of Swin Transformers with enhanced CNN features through a reverse residual module, thereby achieving efficient global feature learning and hierarchical feature extraction. The self-attention mechanism of Transformers leads to the secondary computational complexity related to the sequence length, which significantly increases the computational demand. The structured state space sequence model (SSM) is an effective method to solve the secondary complexity problem of Transformers. The Mamba model is a well-known SSM architecture. Through multi-scale state space aggregation and bidirectional information processing, it achieves linear computational complexity in long sequence modeling. These improvements in efficiency promote the development of remote sensing applications and provide innovative methods for complex scene segmentation, accurate edge detection, and improvement of noise resistance in sea–land imaging. Mamba’s key benefits over CNNs and Transformers include the following: (1) a selection mechanism that dynamically parameterises SSMs to selectively retain pertinent information; (2) a hardware-aware recursive scanning algorithm that substitutes convolutional operations for improved efficiency; (3) near-linear scalability for high-resolution images while preserving modeling performance akin to Transformers. The segmentation capabilities of Mamba have been tested in initial applications across fields such as biomedicine and signal processing, showcasing its competitive performance. Xie et al. [14] used Mamba’s linear complexity global receptive field as the base network to reveal important patterns for evaluating image color aesthetics. Zhang et al. [15] created an Algae Mamba network that integrates small kernel convolution modules to collect local spatial and channel information to facilitate multi-scale processing and enhance generalization ability.

**Figure 1:**
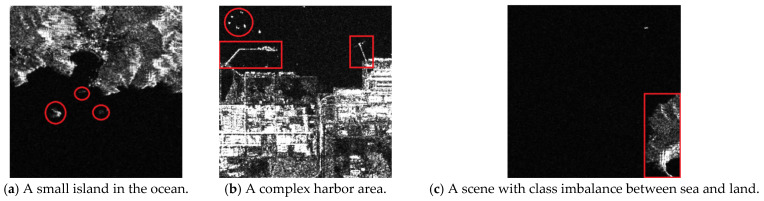
SAR-based remote sensing image of sea–land segmentation. The red regions illustrate the challenges in sea–land segmentation tasks: (**a**) Small targets such as islands and reefs exist in the middle sea–land interface (**b**) The port of the sea–land interface is more complex, with noise ship interference, and there are port objects and port structures similar in colour and texture. (**c**) There is an inter-class imbalance between sea and land.

Despite the promise of deep learning for sea–land segmentation, several challenges persist, as illustrated in Figure 1. Figure 1a it is difficult to achieve accurate segmentation for small targets with irregular shapes in the ocean, such as islands and reefs; Figure 1b complex coastline geometries, such as jagged coasts and estuaries, require finer edge segmentation to improve overall accuracy; and Figure 1c the significant difference in area between land and sea regions in typical images leads to class imbalance, further increasing the difficulty of segmentation.

To address these challenges, this work makes the following key contributions:

(1) We propose a dual-branch encoder architecture integrating ConvNeXtV2 [16] and VMamba [17]. This design synergistically combines ConvNeXtV2’s local feature extraction capability with VMamba’s long-sequence modeling strength, achieving complementary enhancement of local details and global semantics.
(2) We design the Dynamic Multi-Scale Attention (DyMSA) module, which replaces traditional static fusion (e.g., fixed convolution after concatenation) with a dynamic channel-spatial dual-path approach. This addresses the limitations of fixed weighting for deep and shallow features, effectively preserving high-frequency details.
(3) We introduce the Dynamic Weighted Cross-Attention (DyWCA) module, which leverages dynamic weighting and cross-attention to adaptively fuse local features with global semantics. This resolves the issues of inflexible feature integration and edge detail loss inherent in traditional static fusion methods.
(4) The comprehensive study on the publicly available Sentinel-1 SAR dataset [18] and GF-3 SAR sea–land segmentation dataset [19] shows that CVMFusion achieves excellent performance. Compared to existing advanced methods, our method has achieved comprehensive improvements in key indicators, such as MIoU, FgIoU, and F1-score, and has shown significant advantages in detecting small targets and delineating complex boundaries, fully verifying its excellent accuracy and robustness.

## 2. Related Work

Synthetic aperture radar (SAR) images of ocean and land scenes exhibits numerous distinct characteristics. This encompasses intricate and varied backdrops, a minimal percentage of edge pixels necessitating accurate segmentation, intrinsic noise, and an uneven distribution of categories. Consequently, the use of deep learning to sea–land segmentation in remote sensing systems necessitates targeted enhancements and optimizations to overcome these problems. This section examines contemporary research in deep learning-driven picture segmentation from three principal perspectives: the U-Net architecture, attention processes, and multi-branch networks.

### 2.1. Improvements to the U-Net Structure

#### 2.1.1. CNN-Based U-Shape Network for Land and Sea Image Segmentation

The U-Net architecture, which combines a convolutional neural network (CNN) with a U-shaped design, achieves multi-scale feature fusion and maintains spatial information through its symmetric encoder–decoder structure and skip connections. Due to these advantages, U-Net-based architectures have remained a prominent research focus. For instance, R. Li et al. [3] proposed DeepU-Net. This model alters the usual down sampling and up sampling modules to produce a deeper and more efficient CNN, thereby extracting richer feature representations. Shamsolmoali et al. [20] developed the Residual Dense U-Net (RDUNet). This architecture includes densely connected residual blocks into the down sampling and up sampling paths, enabling excellent sea–land segmentation in complicated, high-density remote sensing data.

#### 2.1.2. Transformer-Based U-Shaped Network for Sea and Land Image Segmentation

The integration of the Swin Transformer with the U-shaped architecture utilizes global attention processes and multi-scale feature fusion. The purpose of introducing this module is to enhance the model’s ability to depict complex scenarios and long-range dependencies, thereby offering performance improvements compared to traditional convolutional neural network techniques. This technological improvement shows significant enhancement in the segmentation of remote sensing images. For example, Q. Tong et al. [13] proposed STIRUnet, a novel U-shaped network structure. By leveraging the global modeling capability of the Swin Transformer, it can effectively capture global context and extract local features, making it suitable for sea–land segmentation in complex environments.

#### 2.1.3. VMamba U-Net Model for Image Segmentation

By leveraging the long-range representation capabilities of VMamba’s state space model to replace traditional CNN or Transformer encoders, this approach can achieve higher computational efficiency while maintaining high accuracy. Tapas Kumar Dutta [21] integrated the Mamba-Prior module into the encoder, which addresses the performance gap between general and specific features during pretraining by incorporating prior knowledge of key areas in images into the Segment Anything Model (SAM) encoder, thereby improving segmentation accuracy. Y. Zhang and colleagues [22] developed an Edge-Mix Enhanced Mamba (EM-Mamba) model for kidney segmentation. This model relies on Mamba’s ability to capture long-range dependencies, allowing it to integrate the network’s edge information during the encoding phase while combining these edge features at multiple scales during the decoding phase, thereby achieving more accurate kidney segmentation. In addition, Wu et al. [23] proposed an unsupervised change detection network based on VMamba. This network model introduces a Multi-Scale Feature Decoding (VCFD) module, which uses two weight-shared VMamba encoders to extract multi-scale features from remote sensing images at multiple temporal phases, providing a clearer representation of global context and local details of feature changes.

### 2.2. Improvements in Attention Mechanisms

Sea and land image sets often contain complex scenes, such as islands, harbours and reefs, as well as other small targets. Background interference caused by intrinsic image noise can adversely affect the accuracy of sea and land segmentation. Some researchers therefore focus on improving segmentation accuracy through multi-scale feature fusion and the introduction of an attention mechanism. Xie et al. [24] proposed a two-way global information optimisation network (DGIONet), which uses vertically constructed rectangular strip convolution at different scales to achieve the effect of multi-scale kernel convolution. This relies on extracted multi-scale features and point convolution within the module to achieve a spatial attention mechanism. This effectively improves the network’s attention to sea–land large kernel convolution. This improves the network’s ability to pay attention to large-scale sea and land features and realise the extraction of global and contextual sea and land features. Xing Wang [25] proposed MFB (Mamba Fusion Block), consisting of two parts: one similar to the previous Mamba Block and the other our integrated linear attention and Mamba-like linear attention (MFB). The Mamba Linear Attention (MLLA) improves the model’s performance for point cloud segmentation. Ding et al. [26] designed the LANet, which enhances feature representation by utilising the Plaque Attention Module (PAM) and the Attention Embedding Module (AEM). This achieves superior performance for remote sensing image segmentation.

### 2.3. Improvement of Multi-Branch Network Structure

The primary goal of sea–land segmentation is the accurate delineation of the coastline. Consequently, boundary segmentation accuracy is a key evaluation criterion. To enhance boundary accuracy, some researchers have expanded network architectures by incorporating dedicated edge branches and employing multi-task learning to jointly optimize both edge detection and segmentation tasks. For example, Ji et al. [27] proposed a Dual-Branch Integrated Network (DBENet), which consists of a dense branch and a residual branch. This architecture is designed to extract multi-level features and fuse them using an attention module, thereby improving boundary segmentation accuracy. In a different context, Qi et al. [28] proposed a Mamba-CNN model for remote sensing image scene classification. They introduced HWC-Mamba to perform one-dimensional selective scanning within a 3D image space, thereby establishing a global receptive field. To address Mamba’s deficiency in catching short-range characteristics, a CNN was incorporated. This hybrid architecture improves the model’s feature extraction ability by integrating the global context of Mamba with the local detail acquisition of CNN. Liu et al. [15] introduced the CWmamba model, which utilizes an innovative design that integrates a Mamba block with a CNN-based feature extraction block (BCGF) for the analysis of diachronic (time-series) pictures. During the encoding phase, the model employs Mamba blocks to assimilate global characteristics and BCGF blocks to augment local features. During decoding, multi-level characteristics are included to enhance the model’s total representational capacity.

## 3. Our Method

### 3.1. Network Architecture

We propose a sea–land segmentation network that combines CNN and Mamba architectures to precisely delineate borders in remote sensing imagery.

**Figure 2:**
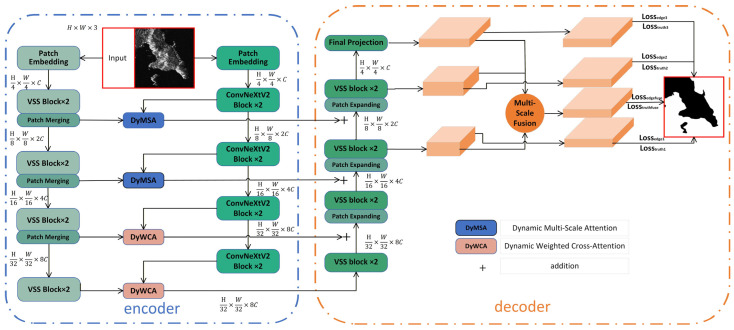
A network architecture for the sea–land separation algorithm is proposed, which is based on a Mamba and CNN-based encoder–decoder architecture, including a dual-branch encoder, MyWCA module, and MyMSA module.

Figure 2 illustrates the comprehensive architecture of our proposed sea–land segmentation network, which adheres to an encoder–decoder framework. The encoder incorporates a ConvNeXtV2 backbone [16] and a VMamba-T network, excluding its fully connected layer.

The ConvNeXtV2 branch represents a modern convolutional architecture that combines the efficiency of a pure CNN with the design philosophy of a Vision Transformer. Unlike traditional CNNs [10,29,30,31], ConvNeXtV2 adopts a deep separable convolution and inverted bottleneck structure, which can effectively capture multi-scale local information while improving model representation ability and maintaining computational efficiency. The ConvNeXtV2 network adopts a four-level feature extraction structure, where each stage gradually reduces spatial resolution while synchronously increasing channel dimensions. This design enables the network to capture delicate texture and edge features in shallow layers, and gradually construct rich semantic representations in deep layers. Finally, the encoder outputs four-scale feature maps with downsampling magnifications of 4, 8, 16, and 32, laying the foundation for subsequent multi-scale feature fusion.

On the other hand, VMamba, as a new generation sequence model, can effectively model long-range dependencies between pixels while maintaining linear computational complexity. Its efficiency stems from its state space model framework, which consists of four stages, each stage consisting of continuous VSS modules and image block merging operations. The process of merging image blocks not only increases channel capacity but also reduces the spatial resolution of feature maps, thereby achieving efficient processing of multi-scale information. The VSS module utilizes the ability of long sequence modeling to directly depict the macro patterns of land–sea distribution, thereby achieving in-depth analysis of coastal environments. The VMamba branch creates feature maps at downsampling ratios of 4, 8, 16, and 32, ensuring spatial alignment with the outputs of ConvNeXtV2. The decoder uses a U-shaped design to effectively combine features from both the Mamba and CNN branches. It includes VSS blocks and patch expander modules that gradually upsample the encoder’s detailed semantic features. We suggest a hierarchical fusion strategy: In the early stages (1–2), the Dynamic Multi-Scale Attention (DyMSA) module uses adaptive channel-spatial weighting through convolutional operations. This module enhances subtle details and spatial relationships within surface-stage features. In later stages (3–4), the Dynamic Weighted Cross-Attention (DyWCA) module uses adaptive cross-attention to combine global context with local semantic characteristics. The features fused at each stage of the dual-branch encoder are integrated with the decoder via U-Net-style skip connections: specifically, the fused features extracted by the dual-branch encoder through the fusion module are concatenated along the channel dimension with the feature maps of the corresponding scale obtained by the decoder through upsampling. This method provides the decoder with both original hierarchical features and enhanced fused representations, thereby ensuring a comprehensive flow of information. The features at different stages and the fused features in the decoder are subject to multi-level loss supervision. The final segmentation result is generated by the output of the three-level fusion decoder, which can accurately outline the sea–land boundary while preserving details and contextual information.

### 3.2. VSS Block

**Figure 3:**
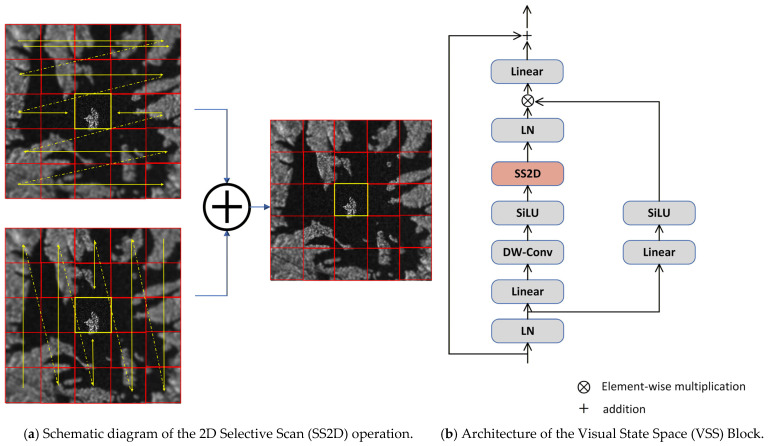
The core components of the VMamba encoder. (**a**) The 2D Selective Scan (SS2D) mechanism establishes long-range dependencies by scanning image patches in four directions (top-left to bottom-right, bottom-right to top-left, horizontal, and vertical). The red box highlights the query patch that initiates correlation with other patches, and the yellow arrows indicate the multi-directional scanning paths along which the query patch interacts with distant patches. (**b**) The detailed structure of the Visual State Space (VSS) Block. It employs a dual-branch design, where one branch projects context and the other performs spatial scanning through the SS2D module, followed by feature fusion via element-wise product and a residual connection.

The Visual State Space (VSS) block [17] is an essential element of VMamba and the pure SSM architecture. It adapts Mamba’s efficient long-range modeling capability to 2D image data within the VMamba framework. As shown in Figure 3, the module utilizes a dual-branch architecture that thoroughly extracts image context through coordinated feature transformation, local enhancement, and global scanning.

The VSS module begins by subjecting the input to layer normalization (LayerNorm), which is subsequently processed through two concurrent pathways: a context projection branch and a spatial scanning branch. The context projection branch enlarges the channel dimension using a linear layer, followed by the application of the SiLU activation function to produce non-linear features. Simultaneously, the spatial scanning branch processes the features via a linear layer, a depthwise separable convolution (DWConv), and the SiLU activation function. This sequence of actions permits the acquisition of local spatial properties, which are subsequently supplied into the core 2D selective scanning module (SS2D). The output from the spatial scanning branch is then normalized by an additional LayerNorm before being processed by the SS2D module. The outputs from both branches are integrated using the Hadamard product and a residual connection, which helps stabilize gradient flow during training. The use of depthwise separable convolutions gives the State Space Model (SSM) a local inductive bias. This solution effectively addresses the original Mamba module’s inherent issue in storing local structural information. The SS2D serves as the main operator within the VSS block. It splits the spatial dependence modeling in two-dimensional images into a four-phase scanning procedure, which comprises scan expansion, selective state-space modeling, and scan amalgamation. The scan expansion phase creates image patches in four orientations: from the top-left to the bottom-right, from the bottom-right to the top-left, and their horizontal and vertical counterparts, providing four one-dimensional sequences. Within selective state-space modeling, each sequence undergoes analysis by the S6 block, which performs data-dependent global modeling. The S6 block augments the S4 block by integrating a selection mechanism that dynamically alters the discretized parameter for input-adaptive information filtering. Afterward, the scan merging step transforms the four output sequences into two-dimensional features. These features are then combined by summation, which reconstructs the original spatial dimensions. This architectural design allows for the creation of global receptive fields with linear complexity. It also preserves spatial relationships by using multidirectional scanning, which helps to reduce structural distortions that can occur with unidirectional serialization.

### 3.3. Dynamic Multi-Scale Attention (DyMSA) Block

Sea–land segmentation in remote sensing data entails intricate textural attributes. Conventional approaches generally integrate deep semantic information and surface texture data through simple concatenation or predefined convolutional operations. However, this static fusion method lacks the ability to dynamically adjust feature importance based on the input data. Consequently, it demonstrates limited adaptability in complex scenarios and frequently omits crucial edge details. In view of these limitations, the Dynamic Multi-Scale Attention (DyMSA) module combines dynamic weighting and a context attention mechanism to achieve adaptive feature fusion. The channel attention mechanism determines the priority of important channels by evaluating the feature significance in the channel dimension. Simultaneously, spatial attention discerns prominent areas by examining the spatial relationships among feature maps. DyMSA integrates attention methods to dynamically collect deep semantic and superficial texture components via weight assignment based on feature significance. This method improves edge and detail retention while ensuring semantic coherence.

**Figure 4:**
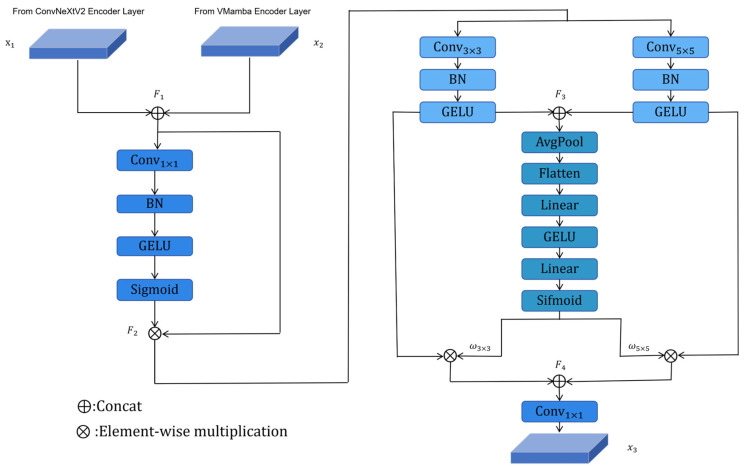
The structure of the proposed DyMSA module, which dynamically fuses multi-scale spatial features and enhances channel-wise information (Refer to Equations (1)–(6) for the computational details).

As shown in Figure 4, The DyMSA module is built on two key components: a channel attention mechanism and a spatial attention mechanism. Initially, the module combines features from the decoder with corresponding features from the encoder’s previous stage. The channel attention mechanism uses a 1 × 1 convolutional compression, a GELU activation function, and a 1 × 1 convolutional restoration process to maintain spatial relationships. After this, it generates channel-specific weights through a sigmoid function, which helps to strengthen semantically important channels. The spatial attention component employs a dual-branch architecture to acquire multi-scale spatial information, extracting local features and contextual data while maintaining spatial resolution. A dynamic weight generator is introduced to adaptively fuse the features of two branches. The generator first aggregates global statistical information through adaptive average pooling. After being flattened, the context vector is passed through two fully connected layers and a sigmoid activation function to generate calibrated dynamic weights, $\omega_{3\times 3},\omega_{5\times 5}$. These weights are expanded dimensionally to match the spatial dimensions of the feature maps. The spatial features are fused through weighted summation, and the final output integrates channel-refined features, adaptively fused multi-scale spatial information, and residual connections. Given input feature maps *x*_1_, *x*_2_ ∈ R^H×W×C^, intermediate states *F*_1_, *F*_2_, $\omega_{3\times 3}$, $\omega_{5\times 5}$, *F*_3_ and the output *x*_3_ are computed as follows:

(1) $$F_{1}=Concat\left(x_{1},x_{2}\right)$$

(2) $$F_{2}=\sigma \left(FC\left(GELU\left(FC\left(F_{1}\right)\right)\right)\right)⊗F_{1}$$

(3) $$F_{3}=Concat\left(GELU\left(BN\left(Conv_{3\times 3}\left(F_{2}\right)\right)\right),GELU\left(BN\left(Conv_{5\times 5}\left(F_{2}\right)\right)\right)\right)$$

(4) $$\omega_{3\times 3},\omega_{5\times 5}=\sigma \left(FC\left(GELU\left(FC\left(Flatten\left(AvgPool\left(F_{3}\right)\right)\right)\right)\right)\right)$$

(5) $$\begin{array}{c} F_{4}=\omega_{3\times 3}⊗\left(GELU\left(BN\left(Conv_{3\times 3}\left(F_{2}\right)\right)\right)\right)+\omega_{5\times 5}⊗\left(GELU\left(BN\left(Conv_{5\times 5}\left(F_{2}\right)\right)\right)\right) \end{array}$$

(6) $$x_{3}=FC\left(F_{4}\right)$$

where *F*_2_ and *F*_3_ are the outputs of the channel and spatial attention sub-modules, respectively; ⊗ and *σ* denote the element-by-element multiplication and Sigmoid activation functions, respectively.

### 3.4. Dynamic Weighted Cross-Attention (DyWCA) Block

**Figure 5:**
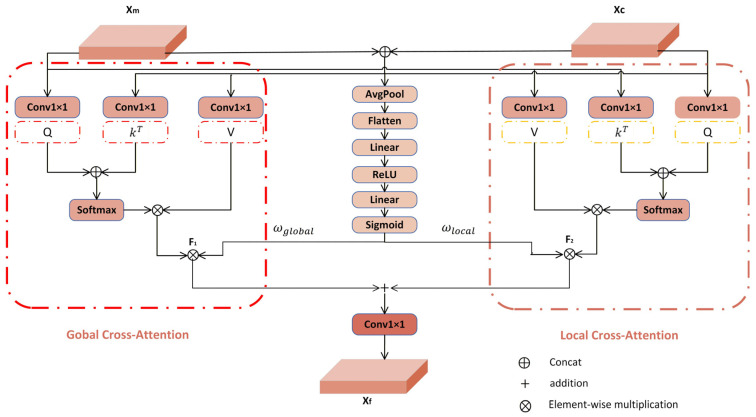
The structure of the proposed DyWCA module, which integrates features from the ConvNeXtV2 and VMamba branches via dynamically weighted cross-attention (Refer to Equations (7)–(11) for the computational details).

In remote sensing sea–land segmentation, convolutional neural networks (CNNs) effectively capture local texture features but often fail to model global contextual information. This limitation leads to suboptimal performance in complex scenes with small targets. In contrast, Mamba-based methods excel at capturing global dependencies but typically lack fine-grained detail preservation. To overcome these combined limitations, we introduce the Dynamic Weighted Cross-Attention (DyWCA) module, which uses features from two different branches (Figure 5). The module incorporates a cross-attention mechanism [32] enhanced with dynamic weighting to effectively fuse features from both branches. It enables feature interaction between the VMamba and ConvNeXtV2 branches through simultaneous global and local cross-attention computation. This design enables joint consideration of global context and local details, significantly enhancing interpretation capability for complex maritime and coastal scenes.

Specifically, the features $X_{c}\in R^{H\times W\times C_{c}}$ from the ConvNextV2 branch and the features $X_{m}\in R^{H\times W\times C_{m}}$ from the VMamba branch are mapped to the query *Q*, the key *K*, and the value matrix V, respectively. Three independent linear layers project the input features into the query, key, and value spaces. The specific formulae are as follows:

(7) $$Q_{c}=W_{c}^{Q}X_{c},K_{c}=W_{c}^{K}X_{c},V_{c}=W_{c}^{V}X_{c}$$

(8) $$Q_{m}=W_{m}^{Q}X_{m},K_{m}=W_{m}^{K}X_{m},V_{m}=W_{m}^{V}X_{m}$$

where H and W denote the width and height of the features, respectively, *C_c_* and *C_m_* denote the number of channels in the CNN and VMamba feature maps, respectively, and C denotes the number of channels in the linear projection, $W_{c}^{Q},W_{c}^{K},W_{c}^{V}\in R^{C\times C_{c}}$ is the learnable weight matrix of the ConvNeXtV2 branch, and $W_{m}^{Q},W_{m}^{K},W_{m}^{V}\in R^{C\times C_{m}}$ is the learnable weight matrix of the VMamba branch. Cross-attention operations are executed between the ConvNeXtV2 and VMamba branches, using global and local calculations to exploit their complementing feature attributes.

The query matrix *Q_m_* from VMamba interacts with the key matrix *K_c_* and value matrix *V_c_* from ConvNeXtV2 to generate the global feature *F*_1_ through global cross-attention. This produces *F*_1_ via cross-attention, with VMamba supplying queries and ConvNeXtV2 providing the correct keys and values.

In a similar manner, the query matrix *Q_c_* from ConvNeXtV2, recognized for its preservation of local details, engages with the key matrix Km and value matrix *V_m_* from VMamba to produce the local feature *F*_2_ ∈ R^H×W×C^.

This design compensates for VMamba’s limited capacity in capturing fine-grained details.

(9) $$F_{1}=softmax(\frac{Q_{m}\cdot K_{c}^{T}}{\sqrt{d_{h}}})\cdot V_{c}$$

(10) $$F_{2}=softmax\left(\frac{Q_{c}\cdot K_{m}^{T}}{\sqrt{d_{h}}}\right)\cdot V_{m}$$

where $d_{h}$ denotes the number of attention heads.

The features from both branches are then concatenated, and their channel dimensions are adjusted via 1 × 1 convolution. A dynamic weight generator assigns adaptive weights to each branch, enhancing informative components while suppressing redundant information.

(11) $$\omega_{global},\omega_{local}=\sigma \left(FC\left(ReLU\left(FC\left(Flatten\left(AvgPool\left(Concat\left(X_{m},X_{c}\right)\right)\right)\right)\right)\right)\right)$$

where $\omega_{global}$ and $\omega_{local}$ represent the dynamic allocation weights of global and local attention, respectively.

### 3.5. Loss Function

Our loss function utilizes multi-tiered supervision for both segmentation and edge sensitivity. The segmentation loss *L_truth_* integrates binary cross-entropy (BCE) [33] and Dice loss [34] to address class imbalance. The edge loss *L_edge_* applies the Sobel operator to extract ground-truth edge masks and computes a Dice loss between the predicted and true edges. This edge supervision enhances coastal detail by encouraging high gradient responses at land–sea boundaries and suppressing blurring. The combined loss guides the network to learn more comprehensive and detailed feature representations.

(12) $$L_{total}=\sum_{i\in S}\gamma_{i}L_{truth}^{i}+\alpha L_{edge}^{i}$$

(13) $$L_{truth}=L_{Dice}+L_{bce}$$

(14) $$L_{edge}=L_{Dice}$$

(15) $$\begin{array}{rr} L_{bce} & =-\sum_{\left(r,c\right)}\left[G\left(r,c\right)log\left(S\left(r,c\right)\right)+\left(1-G\left(r,c\right)\right)log\left(1-S\left(r,c\right)\right)\right] \end{array}$$

(16) $$L_{Dice}=1-\frac{2\left|X\cap Y\right|}{\left|X\right|+\left|Y\right|}$$

where S=(1,2,3,fuse) represents a set of supervision levels, $G\left(r,c\right)$ in {0,1} is the truth value of the pixel point with the coordinate position of $\left(r,c\right)$, and $S\left(r,c\right)$ is the probability that the pixel point with the coordinate position of $\left(r,c\right)$ is predicted to be a saliency target. $\left|X\right|$ and $\left|Y\right|$ denote the true value and prediction, respectively.

The total loss function integrates the contributions of the four supervised points (three levels + fusion layer) and regulates the importance of the depth features through the layer weight coefficients γ_k_, where γ_k_ is set to γ_1_ = 0.4, γ_2_ = 0.6, γ_3_ = 0.8, and γ_4_ = 1.0, so that the high-level fusion features dominate the optimization direction; α1 is set to 0.1 in the early stage of the training to make the pixel-level features dominate the optimization direction; and as the training advances, the gradually increase the weight of the edges to 0.3, so that the network focuses more on the fine optimization of the coastline details after grasping the overall regional distribution.

## 4. Experimental

The model proposed in this paper will be trained and evaluated on publicly available land–sea segmentation datasets with the aim of achieving high-precision land–sea segmentation results.

### 4.1. Experimental Data

We evaluate our method using two distinct sea–land segmentation datasets to ensure robust validation across diverse sensor attributes and geographical regions.

The Sea–Land Segmentation V1.1 dataset, which is publicly available [18], contains 2113 remote sensing images sourced from 21 Sentinel-1 spaceborne SAR scenarios. This dataset covers the coastal regions of southeastern China and Southeast Asia, with a focus on Malaysia and Indonesia. The data underwent thorough preprocessing, including orbit correction, thermal noise removal, radiometric calibration, speckle filtering, and terrain adjustment. To ensure data integrity, each image was carefully reviewed and labeled. Each image has a consistent resolution of 512 × 512 pixels and presents complex scenes featuring diverse land cover types. For this study, a 7:2:1 split was employed, allocating 1479 images for training, 423 for validation, and 211 for testing. To reduce overfitting and improve the model’s ability to generalize, we use data augmentation methods, specifically random horizontal and vertical flipping, along with rotation.

The second dataset, SARSealand V1.0 [19], is a synthetic aperture radar dataset designed for sea–land segmentation tasks. This dataset includes Gaofen-3 images collected using different methods at five different resolutions: 1 m, 3 m, 5 m, 6 m, and 8 m. It contains 2792 labeled images, each showing various boundaries like ports, dams, and natural coastlines. After the initial division, we set aside 1792 images for training, 400 for validation, and 600 for testing. In addition, all images are resized to a standard size of 512 × 512 pixels. The inherent multi-resolution characteristics of the data present considerable difficulties, requiring a thorough assessment of the model’s resilience across diverse data origins, owing to the disparities in spatial attributes. To enhance the model’s ability to define shoreline boundaries, we employ a multi-scale edge detection method to generate edge supervision signals, which are derived from the initial segmentation masks. This methodology computes Sobel gradient responses subsequent to Gaussian blurring at three distinct scales (σ = 1, 2, 3), which are then combined through inverse weighting fusion. The fine scale, with a standard deviation of 1, preserves detailed edge characteristics of small islands and complex ports. In contrast, the coarse scale, with a standard deviation of 3, effectively reduces the inherent speckle noise present in synthetic aperture radar (SAR) images. Furthermore, the medium scale, with a standard deviation of 2, maintains the continuity of prominent coastlines, ultimately producing high-quality edge supervision signals that facilitate improved boundary delineation.

These complementary datasets enable thorough evaluation under varying imaging conditions, sensor characteristics, and geographical contexts, assessing practical applicability in real-world scenarios.

### 4.2. Experimental Configuration

(1) Evaluation metrics: This paper uses the following metrics as the main criteria to evaluate the effectiveness of sea–land segmentation and comprehensively assess the accuracy and performance of various networks: s: F1-score [35], MAE [36], MIoU [37], FgIoU [38], and OA [39].

(17) F1-score=2×Precision×RecallPrecision×Recall

(18) $$MAE=\frac{1}{W\times H}\sum_{x=1}^{W}\sum_{y=1}^{H}\left||S(x,y)-G(x,y)|\right|$$

(19) $$MIOU=\frac{1}{k+1}\sum_{i=0}^{k}\frac{TP_{i}}{FN_{i}+FP_{i}+TP_{i}}$$

(20) $$FgIoU=\frac{\sum_{i=1}^{N_{pos}}\max_{j=1}^{N_{\mathrm{gt}}}IoU(roi_{i},gt_{j})}{\sum_{i=1}^{N_{pos}}1\left\{\max_{j=1}^{N_{\mathrm{gt}}}IoU(roi_{i},gt_{j})>0\right\}}$$

(21) $$OA=\frac{\sum_{i=1}^{k}{TP}_{i}}{\sum_{i=1}^{k}\left({TP}_{i}+{FP}_{i}+{FN}_{i}\right)}$$

Among these, precision refers to the proportion of pixels that the classifier correctly predicts as positive out of all the pixels it predicts as positive. Recall refers to the proportion of pixels that the classifier correctly predicts as positive out of the actual number of positive pixels. W and H denote the width and height of the image, respectively, representing the pixel values at the corresponding positions in the predicted saliency map and the actual ground-truth map. K denotes the number of categories. ${TP}_{i}$ denotes the number of pixels correctly classified into category i. ${FP}_{i}$ denotes the number of pixels incorrectly classified into category i. ${FN}_{i}$ denotes the number of pixels missed in category i. N denotes the total number of positive samples, i.e., objects, in an image and $n_{i}$ denotes the number of actual objects in the image. IoU(i, j) denotes the intersection-over-union ratio between the i-th candidate region and the j-th true object. The indicator function 1{·} returns 1 if the condition inside the brackets is true and 0 otherwise.

Configuration method: The land–sea segmentation model presented in this paper has been implemented using the PyTorch2.4.0 framework and has been trained on a local Linux server running Ubuntu 20.04. The specific software and hardware environment of the local server can be seen in Table 1.

(2) Experimental details: The algorithm used in this paper was set up as follows: the batch size was set to 8, and the AdamW [40] optimiser was used with an initial learning rate of 1 × 10^−4^. The CosineAnnealingWarmRestarts scheduler [41] was used, with a maximum number of iterations of 30, a minimum learning rate of 1 × 10^−5^, and 30 training epochs. The ConvNextV2 and VMamba feature extraction networks were selected as the backbone networks for the algorithm’s encoder component; these networks remove the fully connected layers and include batch normalization layers. The parameters were initialized using weights pre-trained on the ImageNet-1k dataset. For the decoder component, we used the default parameter initialization method provided by the PyTorch deep learning toolkit.

### 4.3. Comparison with Other Algorithms

The proposed sea–land segmentation network was compared with other mainstream segmentation algorithms, including U-Net [42], Deeplabv3+ [43], Unet++ [44], Swin-UNet [45], SegNet [46], VM-UNet [32], VM-UNetV2 [47], UNetV2 [48], and TransFuse [49].To ensure a fair comparison, all baseline models were trained and evaluated under the same experimental setup. All comparison methods used the same data split as described in Section 4.1. During training, consistent data augmentation was applied, including random horizontal flipping, vertical flipping, and rotation. All input images were 512 × 512 pixels. Each model followed the same training details as our model; they were trained for 30 epochs using the AdamW optimizer and CosineAnnealingWarmRestarts scheduler, with an initial learning rate of 1 × 10^−4^ and a batch size of 8. The detailed results of the comparison experiments are shown in Table 2. Table 2 shows how various models compare on the Sea–Land Segmentation V1.1 dataset and SARSealand V1.0 dataset, with the best results highlighted in bold.

**Figure 6:**
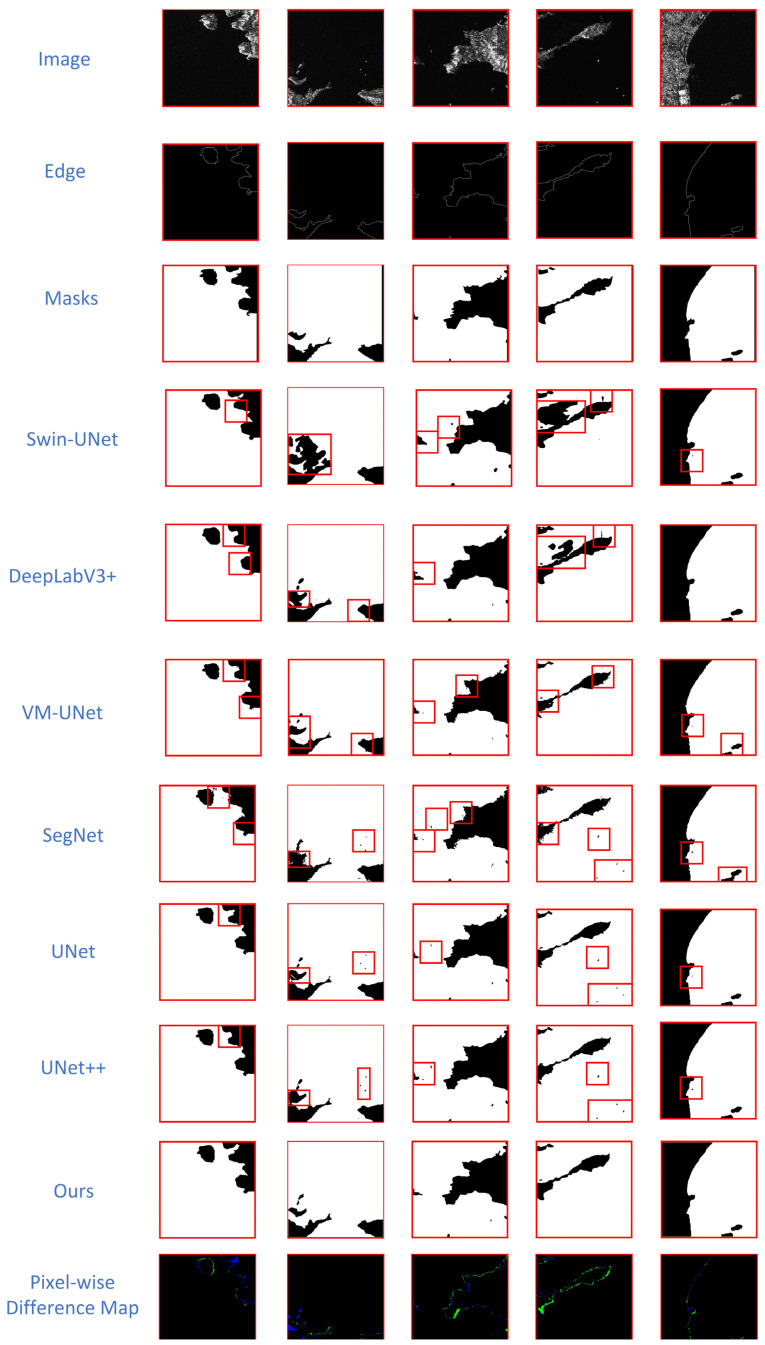
Qualitative comparison of different segmentation models on the Sea–Land Segmentation V1.1 dataset. The proposed CVMFusion produces more accurate boundaries and better identifies small islands (see red boxes) compared to other methods. The Pixel-wise Difference Map shows the disparities between Masks and Ours, where black indicates correctly predicted regions, green represents false positives (predicted as land but the true mask corresponds to ocean), and blue denotes false negatives (true mask corresponds to land but predicted as ocean).

**Figure 7:**
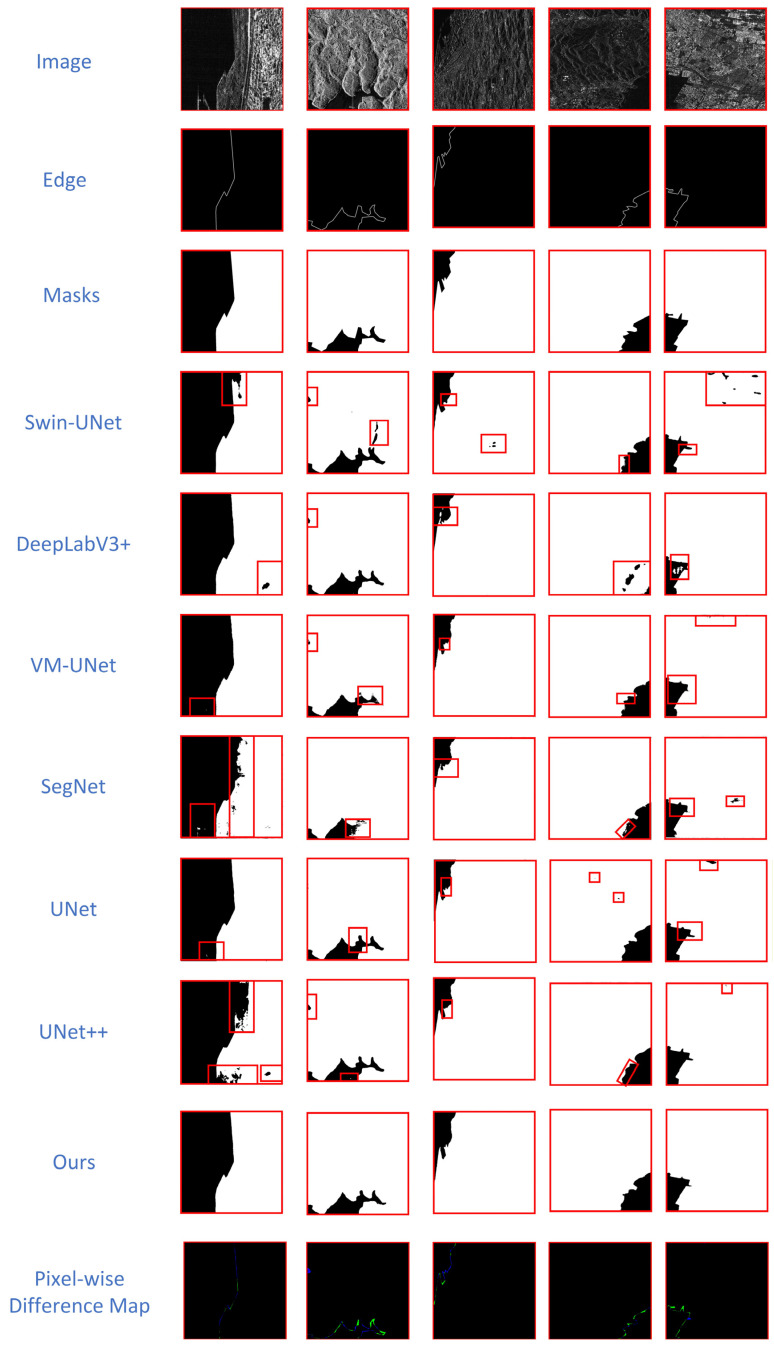
Qualitative comparison on the multi-resolution SARSealand V1.0 dataset. CVMFusion effectively reduces misclassifications in inland areas (see red boxes) demonstrating superior robustness. The Pixel-wise Difference Map shows the disparities between Masks and Ours, where black indicates correctly predicted regions, green represents false positives (predicted as land but the true mask corresponds to ocean), and blue denotes false negatives (true mask corresponds to land but predicted as ocean).

(1) Sea–Land Segmentation V1.1 [18]: We compare CVMFusion against several methods using this dataset. As shown in Table 2, CVMFusion achieves an MAE of 0.0105, an F1-score of 99.03%, an mIoU of 98.05%, an FgIoU of 97.45%, and an OA of 99.70%. These results represent improvements across multiple metrics. In contrast to VM-UNet, a pioneering model utilizing only Mamba, the MAE exhibited a reduction of 0.20%, which suggests improved prediction of edges and fine details. The 0.62% increase in mIoU demonstrates that our attention-focused U-Net architecture enhances the acquisition of global context and the coherence of segmentation. Furthermore, the 0.43% increase in FgIoU indicates improved integrity of target regions and border precision. The combined results show that our hybrid CNN-Mamba architecture effectively captures both global relationships and local details, which leads to better performance than previous methods. Moreover, the qualitative results support the effectiveness of our approach. Figure 6 presents a qualitative comparison of typical scenarios on the Sea–Land Segmentation V1.1 dataset, covering small island detection, complex boundaries, estuarine jagged coastlines, and class-imbalanced scenes. Red circles indicate the differences between segmentation results of various models and ground truth labels. Traditional CNN methods like U-Net and U-Net++ initially exhibit missed detections or fragmented segmentation due to their receptive field limitations, resulting in discontinuous predictions at island edges. While VM-UNet improves global consistency, it excessively smooths small island boundaries, sacrificing sharp geometric features in some local regions. Although MIoU surpasses traditional CNN methods, high-frequency details remain lost. Compared to other models, CVMFusion maintains superior overall contours and edge sharpness across all typical scenarios, achieving precise segmentation even in complex sea–land boundary features and small target scenarios. Finally, we perform pixel-wise difference analysis between masks and our predicted results to further visualize segmentation quality, demonstrating stable accuracy in challenging conditions: our method avoids typical segmentation errors (e.g., incorrect segmentation regions or missed small targets) entirely—these errors are common in other approaches. Only minor pixel deviations exist, with errors concentrated in fine-edge segmentation, indicating room for improvement in our algorithm’s precision handling.

(2) SARSealand V1.0: The dataset results are presented in Table 2. In comparison to VM-UNet, CVMFusion achieves an F1-score improvement of 0.79 percentage points and an MIoU increase of 1.13 percentage points. For the FgIoU metric, CVMFusion reaches 95.85%, which is 1.05 percentage points higher than VM-UNet (94.80%), reflecting improved integrity of target regions and boundary delineation in multi-resolution SAR scenes. This result clearly demonstrates the superiority of CNN-Mamba-based models when compared to other models in the context of SAR sea–land segmentation. Figure 7 illustrates that traditional approaches (U-Net, U-Net++, Swin-UNet, SegNet, and DeepLabV3+) exhibit significant void artifacts in inland regions; furthermore, U-Net, U-Net++, and DeepLabV3+ erroneously categorize vessels as sea in oceanic areas. CVMFusion demonstrates enhanced performance in both terrestrial and maritime regions, exhibiting markedly fewer misclassifications. These approaches have a major flaw: they cannot handle images with different resolutions, which leads to consistent errors. For example, sites far from the coast, where radar signals bounce back weakly, are incorrectly classified as being at sea. At the same time, ships that reflect radar signals strongly in the ocean are mistakenly detected as being on land. Moreover, Swin-UNet and DeepLabV3+ exhibit diminished accuracy near coastlines due to insufficient incorporation of edge information. CVMFusion attains good overall accuracy; nonetheless, it has small limitations in edge refinement, resulting in a slight smoothing of fine coastline protrusions and indentations. This is probably attributable to the edge supervision mechanism, which promotes more seamless border extraction. Finally, we still perform a pixel-wise difference between the mask and our method’s predicted map to further visualize the segmentation quality on the SARSealand V1.0 dataset: Our method aligns almost perfectly with the ground truth, with only minor false detections (green areas) at some edges and virtually no missed detections. It outperforms the comparative models in terms of error ratio, boundary precision, and region adaptability, demonstrating high pixel-level consistency between predicted results and the true mask, achieving ideal segmentation quality.
(3) To quantitatively evaluate whether the performance difference between CVMFusion and comparative methods has statistical reliability, we conducted Wilcoxon sign rank test on CVMFusion and three typical methods representing CNN, Transformer, and Mamba techniques based on MIoU index. As shown in Table 3, on the Sea–Land Segmentation V1.1 dataset, the improvement of CVMFusion compared to U-Net++ is statistically significant, reaching edge significance compared to Swin Unet method. Although the difference with VM-UNet does not reach the traditional significance level, it still shows clear performance advantages in MIOU metrics.

This indicates that on relatively simple and small sample datasets, the performance of advanced methods is close to saturation, and small overall performance improvements may be difficult to achieve statistical significance. On the more challenging SARrealand V1.0 multi-resolution dataset, CVMFusion exhibits clearer statistical significance: our methods all achieve statistical significance. This proves that the architecture advantage of CVMFusion is stable in more complex scenarios.

### 4.4. Ablation Experiment

In this section, we conducted ablation studies to evaluate the effectiveness of each component involved in CVMFusion in a comprehensive manner. Specifically, we verified and analyzed the impact of the dual-branch encoder and the DyMSA and DyWCA modules on the Sea–Land Segmentation V1.1 dataset and sea–land SARSealand V1.0 dataset, and conducted qualitative analysis in combination with multi-level feature visualization.

(1) Ablation experiments on the importance of encoders:

An in-depth exploration of how different encoder components impact the overall performance of the model by sequentially modifying key encoder parts to quantify their influence on image segmentation task results.

The control groups are set as follows: (1) using only the ConvNextV2 network as the encoder; (2) using only the VMamba network as the encoder; (3) using both the ConvNextV2 and VMamba networks as a dual-branch encoder.

Table 4 shows the results of the various ablation methods on the Sea–Land Segmentation V1.1 dataset and SARSealand V1.0 dataset. It indicates that combining shallow and deep features allows the model to use information from different branch encoders, improving the network’s segmentation performance.

**Figure 8:**
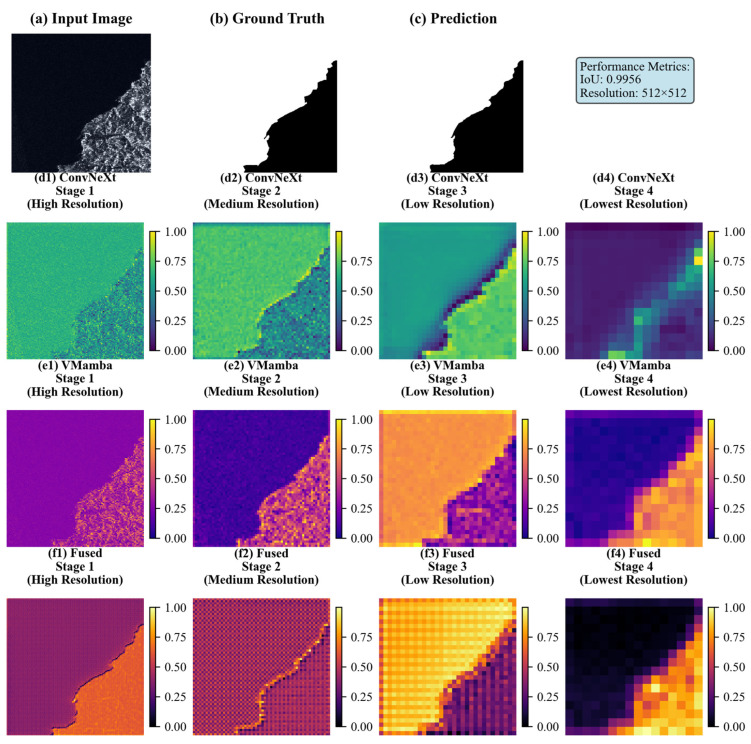
(**a**) Input SAR image; (**b**) Ground truth; (**c**) Prediction result; (**d1**–**d4**) Four-stage feature responses of the ConvNeXtV2 branch, using the viridis color scheme, showing a continuous gradient from blue → green → yellow, with color values reflecting the response intensity of features extracted at each stage of ConvNeXtV2; (**e1**–**e4**) Four-stage feature distributions of the VMamba branch, using the plasma color scheme, showing a continuous gradient from purple → pink → orange → yellow, with color values reflecting the response intensity of features extracted at each stage of VMamba; (**f1**–**f4**) Feature maps after fusion, using the inferno color scheme, showing a continuous gradient from black → red → orange → yellow, with color values reflecting the response intensity after feature fusion at each stage.

Table 4 presents quantitative results that validate the effectiveness of our encoder on the Sea–Land Segmentation V1.1 dataset. In terms of global metrics, the Mean Intersection over Union (MIoU) increased by 0.95% over the single-branch ConvNeXtV2 and by 0.62% over the single-branch VMamba. This result confirms that the dual-branch architecture enhances global segmentation accuracy. The overall accuracy (OA) reached 99.70%, significantly outperforming ConvNeXtV2 (98.50%) and VMamba (99.23%). For foreground object and boundary accuracy, the dual-branch architecture achieved an FgIoU of 97.45%, which is 0.11% higher than that of the single-branch VMamba. The F1-score of the dual-branch architecture also exceeded those of ConvNeXtV2 and VMamba by 0.65% and 0.34%, respectively. Figure 8 visualizes the hierarchical features across different stages on the Sea–Land Segmentation V1.1 dataset, highlighting the distinct characteristics of each module at various resolutions. At high resolution (Figure 8(d1)), the ConvNeXtV2 branch exhibits gradient distributions of coastal scatter textures and near-shore coarse scatter clusters. This effectively encodes the fine-grained scattering characteristics of SAR data, such as the heterogeneity along jagged coastlines and the speckled noise textures in small reef areas. This demonstrates the inherent advantage of the hierarchical convolution-based feature encoding mechanism in capturing local SAR details, thereby providing ‘fine-grained feature anchors’ for sea–land boundary segmentation. However, at medium-to-low resolutions (Figure 8(d2–d4)), the cross-domain semantic consistency between offshore and land areas deteriorates markedly, despite the hierarchical aggregation of semantic information in the feature maps. This exposes the inherent limitation of convolutional architectures in modeling long-range dependencies. In contrast, the VMamba branch shows a relatively smooth distribution at high resolution (Figure 8(e1)) and has a weaker response to local SAR scattering details than ConvNeXtV2. This is due to its VSSM-based global semantic-priority modeling mechanism, which achieves holistic perception across vast sea–land domains in the initial stage. Conversely, at medium-to-low resolutions (Figure 8(e2–e4)), the sea and land regions show strong discriminative color differentiation (e.g., deep purple for ocean and orange-yellow for land) and exhibit sharp global semantic boundaries. This highlights the model’s advantage in modeling long-range dependencies, enabling effective aggregation of cross-regional semantic information in large SAR scenes, such as the backscatter statistical patterns over extensive land areas.

**Figure 9:**
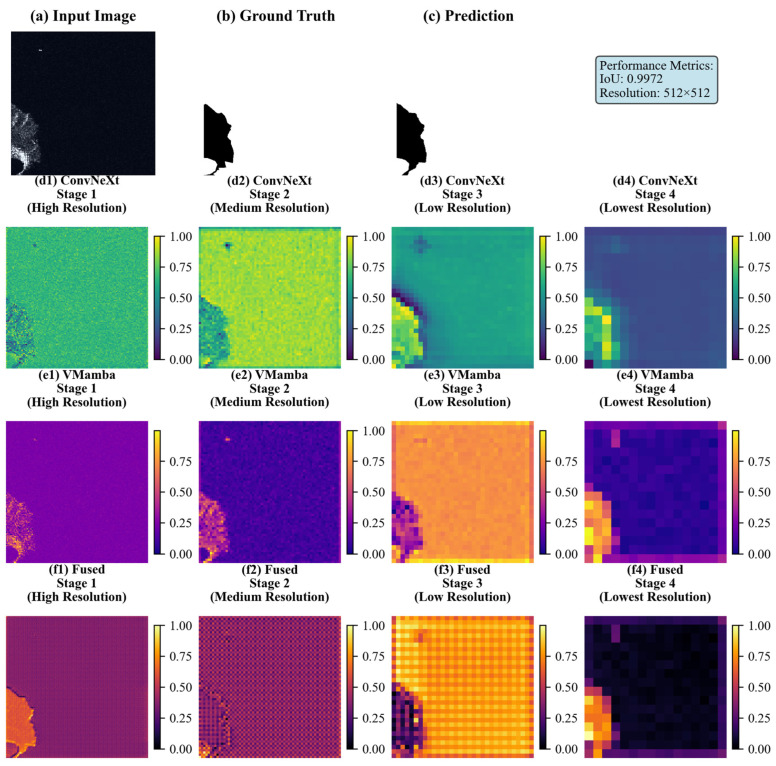
(**a**) Input SAR image; (**b**) Ground truth; (**c**) Prediction result; (**d1**–**d4**) Four-stage feature responses of the ConvNeXtV2 branch, using the viridis color scheme, showing a continuous gradient from blue → green → yellow, with color values reflecting the response intensity of features extracted at each stage of ConvNeXtV2; (**e1**–**e4**) Four-stage feature distributions of the VMamba branch, using the plasma color scheme, showing a continuous gradient from purple → pink → orange → yellow, with color values reflecting the response intensity of features extracted at each stage of VMamba; (**f1**–**f4**) Feature maps after fusion, using the inferno color scheme, showing a continuous gradient from black → red → orange-yellow, with color values reflecting the response intensity after feature fusion at each stage.

Table 4 presents the quantitative results, validating the effectiveness of our encoder on the SeaLand V1.0 dataset. In terms of global metrics, the Mean Intersection over Union (MIoU) increased by 0.81% over the single-branch ConvNeXtV2 and by 0.52% over the single-branch VMamba. The FgIoU was 0.47% higher than that of the single-branch VMamba. Moreover, the F1-score exceeded those of ConvNeXtV2 and VMamba by 0.72% and 0.43%, respectively. Figure 9 visualizes the hierarchical features across different processing stages on the SARSealand V1.0 dataset. At the high-resolution stage (Figure 9(d1)), the ConvNeXtV2 branch exhibits localized gradient variations in the scattering texture of SAR target regions (e.g., the left landmass), thereby accurately capturing the local scattering characteristics of SAR data. This provides “fine-grained feature support” for identifying small SAR targets and complex terrain features. However, during semantic aggregation at medium-to-low resolutions, the cross-region semantic consistency across large SAR scenes is not adequately maintained. This is due to limitations in the convolutional architecture’s ability to model long-range dependencies. At the high-resolution stage (Figure 9(e1)), the VMamba branch exhibits a global response pattern for the left-hand landmass in the SAR image. This demonstrates its advantage in early global semantic perception for large-scale SAR scenes, as it comprehensively recognizes the overall distribution pattern of extensive land areas. At the medium-to-low resolution stages (Figure 9(e2–e4)), the target region exhibits a high degree of structural differentiation from the background, evidenced by the sharp boundary between the orange-yellow terrestrial clusters and the purple oceanic clusters. Given the inherent characteristics of SAR data, such as large scene coverage and strong scattering heterogeneity, the long-range dependency modeling capability of VMamba effectively aggregates semantic information across regions.

In summary, the visualization results from both SAR datasets mechanistically demonstrate the complementary relationship between “fine-grained scattering features” and the “global semantic hierarchy” in the dual-branch encoder: The ConvNeXtV2 branch, leveraging its hierarchical convolutional architecture, excels at encoding local fine-grained scattering features in SAR imagery (e.g., coastal textures and small-target scattering heterogeneity). It offers detailed feature anchors, which are useful for segmentation. In contrast, the VMamba branch, which uses a linear state-space model, focuses on the overall meaning in SAR data. This approach provides “global contextual constraints” to help with segmentation. This combined approach allows the dual-branch encoder to effectively distinguish between different characteristics across a range of sizes and semantic levels. This specific capability is especially useful in the unique context of synthetic aperture radar (SAR). SAR is characterized by speckle noise, feature differentiation that is mainly based on backscatter, and significant semantic relationships that span long distances. These findings confirm the technical efficacy of the proposed dual-branch encoder for SAR land–sea segmentation tasks.

(2) Effectiveness of the DyMSA and DyWCA modules: These modules are key components of CVMFusion. The DyMSA module uses dynamic weight allocation of channel-spatial attention to enhance interactions between dimensions, helping to generate more accurate segmentation masks. Meanwhile, the DyWCA module enhances fine-grained features and global information by allocating weights dynamically to cross-attention between features from the ConvNextV2 branch and the VMamba branch.

The control group is set up as follows: (1) CVMFusion is set up as the baseline network without the DyMSA and DyWCA modules; (2) the baseline is augmented with the DyMSA module; (3) the baseline is augmented with both the DyMSA and DyWCA modules.

Table 5 presents the ablation results. We first establish a baseline by removing both the DyMSA and DyWCA modules from CVMFusion. Progressively incorporating DyMSA and DyWCA leads to consistent improvements, with the complete model achieving the best performance across all metrics.

Figure 8 and Figure 9 demonstrate that for the Sea–Land Segmentation V1.1 dataset, the fusion features from the shallow high-resolution stage (f1) exhibit a pronounced advantage in “detail preservation and local semantic alignment”. Specifically, these fused features inherit the ConvNeXtV2 branch’s capability for fine-grained encoding of gradient variations along jagged coastlines. Furthermore, through the dynamic multi-scale weight allocation of DyMSA, they integrate local semantic awareness (e.g., of small-scale sea–land boundaries) from the VMamba branch. This collaborative approach effectively addresses the problem of “isolated details and ambiguous semantics” that is characteristic of the single-branch ConvNeXtV2 architecture. When compared to the feature maps derived from the single-branch ConvNeXtV2 at the f1 stage, the merged features demonstrate a marked improvement in detail-semantic correlation. This is particularly apparent in the improved coherence between dispersed textures within coastal sub-regions and their corresponding land–sea semantics. As the process advances to the medium-resolution stage (f2), the fused features maintain a considerable degree of detail during semantic aggregation. The scattering characteristics of micro-reefs remain distinct, notwithstanding the reduction in resolution. This is achieved through DyMSA’s dynamic activation of “multi-scale detail channels”, which serve to diminish extraneous background noise while simultaneously amplifying authentic detail signals. As processing progresses to the deep low-resolution stages (f3, f4), the fused features at stage f3 maintain the VMamba branch’s depiction of borders, marked by the differentiation between deep ocean (purple clusters) and terrestrial regions (orange-yellow clusters). The DyWCA cross-branch attention mechanism efficiently integrates scattering signals from isolated small targets in the wide sea, derived from the ConvNeXtV2 branch, into the global semantic framework. This mitigates a significant drawback of the single VMamba branch, specifically its propensity for “semantic coherence at the expense of detail loss”. In comparison to the feature map from the single-branch VMamba at the f3 stage, the amalgamated features exhibit significantly improved response signals for tiny targets in remote maritime areas. This unequivocally illustrates the effectiveness of cross-branch detail augmentation. During the final f4 stage (the lowest resolution), DyWCA dynamically assigns weights to the semantic contributions from both branches. This weighting method ensures that integrated features maintain consistency in broad sea–land semantics while simultaneously preserving scattering heterogeneity at the interface. Consequently, this approach yields a high-level feature representation for the ultimate segmentation task that is characterized by both global coherence and local intricacy.

The SARSealand V1.0 dataset, which comprises composite land–sea-target scenes, illustrates that fusion features derived from the shallow high-resolution stage (f1) significantly enhance encoding accuracy for micro-scale targets. The objectives include “corner reflector scattering from terrestrial structures” and “scatter spots from maritime boats”. This enhancement is achieved as DyMSA dynamically captures scattering information within target areas through its multi-scale windows. The procedure aligns with the local target semantics provided by the VMamba branch, which delineates features such as the scattering disparities between maritime vessels and the oceanic environment. This collaborative approach mitigates the issue of “isolated target details susceptible to noise interference” that is inherent in the single-branch ConvNeXtV2 architecture. At the medium-resolution semantic abstraction level (f2), the integrated features maintain a clear distinction between “port facility scatter clusters” and the surrounding seawater boundaries. This demonstrates a more sophisticated detail-semantic correlation compared to the independent utilization of either branch. Advancing to the deep low-resolution phases (f3, f4), the cross-branch semantic interaction mechanism of DyWCA effectively resolves the conflict between significant semantic ambiguity and the preservation of small-target details. At the f3 stage, the integrated features accurately define broad land–sea semantics, as demonstrated by the global distinction between the left landmass and the right ocean. The cross-attention mechanism simultaneously incorporates ConvNeXtV2’s local scattering patterns into the global semantic structure, thereby enabling the localization of complex targets, even at reduced resolutions. In the final F4 stage, the fusion features improve both global semantic understanding and the anchoring of critical details. The fusion module employs a tiered dynamic mechanism: DyMSA operates at superficial, high-to-medium resolution levels, while DyWCA functions at profound, low-resolution layers. This design effectively mirrors the characteristics of SAR data, which exhibits both scattered information and significant semantic interdependencies.

Specifically, DyMSA achieves semantic enhancement of fine-grained scattering details via multi-scale dynamic weight activation. Conversely, DyWCA performs detail supplementation within a global semantic framework through cross-branch attention. The synergistic operation of these two modules enables the fusion features to consistently demonstrate advantages in both detail preservation and semantic clarity across all resolution stages in the two SAR datasets. This synergy provides the core feature support necessary for enhancing final segmentation performance, thereby validating the technical efficacy of the fusion module at a mechanistic level.

(3) Ablation experiments on the effectiveness of multi-level supervision and edge loss.

This paper uses the Dice loss and cross-entropy loss functions to supervise true value and edge losses at different scales. The control groups set up in this paper are as follows:

Supervise only the true value loss in the last layer of the decoder; (2) supervise both the true value and edge loss in the last layer of the decoder; (3) multi-level supervision of the ground truth and edge loss, but with α fixed at 0.1; and (4) multi-level supervision of the ground truth and edge loss, with the α weight increased by 0.1 every 10 training rounds.

As can be seen in Table 6, combining the edge loss function increased the mIoU from 0.9736 to 0.9734. Furthermore, the multi-level supervision mechanism improved the segmentation accuracy further, reaching an mIoU of 0.9805. This represents the best segmentation accuracy achieved by the network proposed in this paper, proving the effectiveness of the multi-level supervision and edge loss mechanisms.

### 4.5. Efficiency Evaluation

In this paper, we report the computational complexity of our proposed method, encompassing both parameter count (Params) and floating-point operations (FLOPs), as shown in Table 7. Relative to competing approaches, our design demonstrates acceptable computational efficiency, albeit without achieving state-of-the-art inference speed. Additionally, we investigate the scalability of alternative methods by way of a case study: modifying the encoder–decoder configuration of the baseline VM-UNet from (2,2,2,2-2,2,1) to (2,2,9,2-2,9,2,2) resulted in concurrent increases in Params and FLOPs alongside a measurable degradation in model performance. These findings suggest that mere depth augmentation of the network architecture may not yield performance gains for the task of sea–land segmentation in remote sensing imagery. Furthermore, as evidenced by the tabulated results, the fusion module proposed herein contributes effectively to network performance improvement. However, this marginal enhancement is attained at the expense of a substantial increase in model parameters—a limitation of the present work. We hypothesize that this constraint arises from the non-significant synergistic effect between multi-scale CNN-derived local features and global semantic features learned via the hierarchical Mamba structure. Accordingly, a core direction for future research will focus on developing efficient fusion mechanisms to enable more impactful integration of local detail features and global semantic representations.

## 5. Discussion

In this work, we propose CVMFusion: a model that leverages two advanced network architectures, ConvNeXtV2 and VMamba, as its encoders. Qualitative investigation indicates that the CNN-based ConvNeXtV2 encoder proficiently extracts spatial characteristics, including shoreline edges and textures; however, its constrained receptive field of convolution limits its capacity to collect global features. Conversely, the VMamba encoder based on SS2D captures global dependencies of images with linear complexity, but its lack of the local inductive bias of CNNs makes it less capable of capturing image details. In the task of sea–land segmentation in remote sensing images, meticulous management of coastlines and the global coherence of coastlines are paramount. Therefore, we propose two dynamic feature fusion modules: DyMSA and DyWCA, which act at different stages of the dual-branch encoder and dynamically integrate the features extracted at each stage of the dual-branch encoder, achieving adaptive fusion of fine-grained local perception and long-range context modeling.

This paper selects two datasets processed from different types of multi-view images at different distances from the ground, the Sea–Land Segmentation V1.1 dataset (Sentinel-1) and SARSealand V1.0 (GF-3), as benchmarks. The main reason for this is to evaluate the generalization and robustness of the proposed method. A large number of experiments also reveal the generalization of our method in the task of remote sensing sea–land segmentation. Additionally, we conduct a series of ablation experiments to evaluate the effectiveness of the components of the CVMFusion network. In the first and second stages of the encoder, more emphasis is placed on capturing the detailed information of the image. Therefore, we design the DyMSA module based on convolution to take advantage of the local perception of convolution and enhance the cross-dimensional interaction of the features extracted by the ConvNeXtV2 encoder and VMamba encoder in the early stages in terms of channels and space. In the third and fourth stages of the encoder, the later fusion focuses on constructing semantic information. Therefore, we design the DyWCA module based on cross-attention to take advantage of the global modeling ability of the attention mechanism and enhance the integration of the features extracted by the ConvNeXtV2 encoder and VMamba encoder in the third and fourth stages in terms of global semantics. The entire process achieves complementary advantages of CNN and Mamba, and the experimental results prove the effectiveness of the fusion design. Although CVMFusion achieves excellent performance in segmentation accuracy, its dual-branch architecture and dynamic fusion modules also bring higher computational complexity and parameter quantity. This, to some extent, affects the inference efficiency of the model, which is a drawback of our work. We believe the main reason lies in the design of the fusion module and the increase in parameters brought by the dual-branch encoder. In future work, we will focus on researching how to design more efficient fusion modules to replace complex attention mechanisms and lightweight CNN-Mamba segmentation models.

## 6. Conclusions

This research examines significant obstacles in remote sensing sea–land segmentation: indistinct, intricate coastline boundaries and the difficulty in detecting small-scale targets. We propose CVMFusion, a dual-branch segmentation network based on multi-scale feature fusion. The ConvNeXtV2-VMamba dual-branch encoder achieves complementary feature enhancement: ConvNeXtV2 preserves local geometric details like coastline textures through hierarchical convolutions, while VMamba captures long-range dependencies via sequence modeling to represent macro-level sea–land distributions. In the decoder, we design two novel modules: DyMSA and DyWCA. DyMSA enables intra-branch feature fusion through dynamic channel-spatial attention weighting. DyWCA facilitates inter-branch fusion via dynamically weighted cross-attention. They create a bidirectional guidance channel for feature optimization that operates at both micro and macro levels. Experiments show that CVMFusion achieves outstanding performance on the Sea–Land Segmentation V1.1 dataset, with ablation studies validating each module’s contribution. Despite its performance, CVMFusion has limitations: (1) the dual-branch architecture increases computational complexity, limiting real-time application; (2) generalization and cross-domain transfer need improvement; (3) adaptive optimization requires enhancement. Future work will explore lightweight architectures, cross-domain transfer learning, and multi-task frameworks to improve efficiency and practicality. These advancements could enable applications in marine disaster warning systems and smart port management.

## Figures and Tables

**Table 1:** Experimental software and hardware configuration table.

Hardware Configuration			
CPU	GPU	Memory	Disk
Intel(R)Xeon(R)Platinum 8260 CPU@2.30 GHz	NVIDIA GeForce RTX 3090	86 GB	Samsung SSD 870
**Software configuration**			
Operating system	NVIDIA drive	CUDA	Acceleration library
Ubuntu 20.04	525.105.175	11.8	cuDNN8
**Development environment**			
Deep Learning Framework	Programming languages	Algorithm library	GCC compiler
Pytorch = 2.4.0	Python = 3.10	Numpy = 1.220	Gcc 7.5.0

**Table 2:** Comparative experimental results of each model on the Sea–Land SegmentationV1.1 and SARSealand V1.0.

Dataset	Model Category	MAE	F1-Score (%)	MioU (%)	FgIoU (%)	OA (%)
Sea–Land Segmentation V1.1	UNet	0.0185	97.84	96.09	94.40	98.21
	SegNet	0.0127	98.28	96.91	95.86	99.07
	DeepLabV3+	0.0164	98.56	96.77	95.72	96.50
	TransFuse	0.0144	98.42	96.83	96.71	99.06
	Swin-UNet	0.0119	98.13	97.14	96.74	99.38
	UNetV2	0.0135	98.52	97.06	97.01	98.96
	VM-UNet	0.0125	98.61	97.43	97.02	99.16
	VM-UNetV2	0.0132	98.41	97.10	96.68	99.13
	UNet++	0.0141	98.89	97.21	96.27	99.05
	**CVMFusion**	**0.0105**	**99.03**	**98.05**	**97.45**	**99.70**
SARSealand V1.0	UNet	0.0360	96.19	94.95	94.76	96.40
	SegNet	0.0371	94.34	93.05	92.84	95.41
	DeepLabV3+	0.0289	96.56	94.57	94.12	96.11
	TransFuse	0.0352	96.52	95.30	95.08	97.06
	Swin-UNet	0.0301	96.10	94.95	94.04	96.99
	UNetV2	0.0342	96.78	95.03	94.72	96.54
	VM-UNet	0.0378	97.34	95.15	94.80	96.82
	VM-UnetV2	0.0362	97.06	95.42	95.14	97.09
	UNet++	0.0298	96.21	95.06	94.89	97.02
	**CVMFusion**	**0.02** **46**	**98.13**	**96.28**	**95.85**	**97.75**

**Table 3:** Wilcoxon signed-rank test results of CVMFusion and critical baseline on the Sea–Land SegmentationV1.1 and SARSealand V1.0 (* represents significant statistical significance).

Methods	Sea–Land Segmentation V1.1	SARSealand V1.0
U-Net++	*p* = 0.0017 *	*p* = 0.000007 *
Swin-Unet	*p* = 0.067	*p* = 0.036 *
VM-UNet	*p* = 0.132	*p* = 0.047 *

**Table 4:** Ablation experiment on the impact of encoders on the Sea–Land SegmentationV1.1 and SARSealand V1.0.

Dataset	Encoder Group	MAE	F1-Score (%)	MioU (%)	FgIoU (%)	OA (%)
Sea–Land Segmentation V1.1	ConvNextV2	0.01449	98.38	97.10	97.42	98.50
	VMamba	0.0121	98.69	97.43	97.34	99.23
	ConvNextV2+VMamba	0.0105	99.03	98.05	97.45	99.70
SARSealand V1.0	ConvNextV2	0.0305	97.41	95.47	95.22	97.05
	VMamba	0.0280	97.70	95.76	95.38	97.22
	ConvNextV2+VMamba	0.0259	98.13	96.28	95.85	97.75

**Table 5:** Ablation experiments on the impact of DyMSA and DyWCA on the Sea–Land SegmentationV1.1 and SARSealand V1.0.

Dataset	Experiment Group	MAE	F1-Score (%)	MioU (%)	FgIoU (%)	OA (%)
Sea–Land Segmentation V1.1	Baseline	0.0142	98.38	97.42	96.50	99.08
	+DyMSA	0.0119	98.94	97.63	97.30	99.21
	+DyMSA+DyWCA	0.0105	99.03	98.05	97.45	99.70
SARSealand V1.0	Baseline	0.0306	97.26	95.42	94.68	96.40
	+DyMSA	0.0287	97.70	96.14	95.30	97.18
	+DyMSA+DyWCA	0.0259	98.13	96.28	95.85	97.75

**Table 6:** Ablation experiments on the impact of multi-level supervision and edge loss on the Sea–Land SegmentationV1.1 and SARSealand V1.0 (“✓” indicates the strategy is enabled; “✗” indicates the strategy is not enabled.).

Dataset	Experiment Number	Supervision Strategy	Edge Supervision	α Strategy	F1-Score (%)	MIoU (%)
Sea–-Land Segmentation V1.1	(1)	Only the last layer is true	✗	✗	98.04	96.85
	(2)	Only the last layer of truth values + edges	✓	0.1	98.45	97.14
	(3)	Multi-level truth value	✗	✗	98.24	97.02
	(4)	Multi-level truth value + edges	✓	0.1	98.79	97.48
	(5)	Multi-level truth value + edges	✓	Incremental (+0.1/10 epoch)	99.03	98.05
SARSealand V1.0	(1)	Only the last layer is true	✗	✗	96.92	95.08
	(2)	Only the last layer of truth values + edges	✓	0.1	97.20	95.41
	(3)	Multi-level truth value	✗	✗	97.01	95.52
	(4)	Multi-level truth value + edges	✓	0.1	97.80	96.05
	(5)	Multi-level truth value + edges	✓	Incremental (+0.1/10 epoch)	98.13	96.28

**Table 7:** Efficiency Evaluation on the Sea–Land SegmentationV1.1 and SARSealand V1.0.

Model	Params (M)	Flops (G)	The Sea–Land Segmentation V1.1	SARSealand V1.0
			MIoU (%)	MIoU (%)
UNet(ResNet50)	32.52	43.83	96.09	94.95
UNet(ResNet101)	51.51	62.33	96.15	94.42
UNet++(ResNet50)	48.99	230.25	97.21	95.06
UNet++(ResNet101)	67.98	249.75	97.11	94.59
DeepLabV3+(ResNet50)	26.68	36.90	96.77	94.57
DeepLabV3+(ResNet101)	45.67	56.41	97.25	94.12
VM-UNet	27.42	16.45	97.43	95.15
VM-UNet(2,2,9,2-2,9,2,2)	38.28	30.33	97.27	94.66
CVMFusion(-DyMSA-DyWCA)	43.71	29.74	97.42	95.47
CVMFusion(-DyWCA)	45.90	44.04	97.63	95.76
CVMFusion	51.25	46.61	98.05	96.28

## Data Availability

The original data presented in the study are openly available in “GEOMATICS AND INFORMATION SCIENCE OF WUHAN UNIVERSITY” doi: 10.13203/j.whugis20210078 and “IEEE Geoscience and Remote Sensing Letters” doi: 10.1109/LGRS.2024.3461751.
